# The quiet quitting scale: Development and initial validation

**DOI:** 10.3934/publichealth.2023055

**Published:** 2023-10-17

**Authors:** Petros Galanis, Aglaia Katsiroumpa, Irene Vraka, Olga Siskou, Olympia Konstantakopoulou, Ioannis Moisoglou, Parisis Gallos, Daphne Kaitelidou

**Affiliations:** 1 Clinical Epidemiology Laboratory, Faculty of Nursing, National and Kapodistrian University of Athens, Athens, Greece; 2 Department of Radiology, P. & A. Kyriakou Children's Hospital, Athens, Greece; 3 Department of Tourism Studies, University of Piraeus, Piraeus, Greece; 4 Center for Health Services Management and Evaluation, Faculty of Nursing, National and Kapodistrian University of Athens, Athens, Greece; 5 Faculty of Nursing, University of Thessaly, Larissa, Greece; 6 Faculty of Nursing, National and Kapodistrian University of Athens, Athens, Greece

**Keywords:** instrument, scale, quiet quitting, validation, employees

## Abstract

**Introduction:**

COVID-19 pandemic causes drastic changes in workplaces that are likely to increase quite quitting among employees. Although quiet quitting is not a new phenomenon, there is no instrument to measure it.

**Objective:**

To develop and validate an instrument assessing quiet quitting among employees.

**Methods:**

We identified and generated items through an extensive literature review and interviews with employees. We carried out the content validity by content experts and we calculated the content validity ratio. We checked face validity by conducting cognitive interviews with employees and calculating the item-level face validity index. We conducted exploratory and confirmatory factor analysis to investigate the quiet quitting scale (QQS) factorial structure. We checked the concurrent validity of the QQS using four other scales, i.e., Copenhagen burnout inventory (CBI), single item burnout (SIB) measure, job satisfaction survey (JSS) and a single item to measure turnover intention. We estimated the reliability of the QQS measuring Cronbach's alpha, McDonald's omega, Cohen's kappa and intraclass correlation coefficient.

**Results:**

After expert panel review and item analysis, nine items with acceptable corrected item-total correlations, inter-item correlations, floor and ceiling effects, skewness and kurtosis were retained. Exploratory factor analysis extracted three factors, namely detachment, lack of initiative and lack of motivation, with a total of nine items. Confirmatory factor analysis confirmed this factorial structure for QQS. We found statistically significant correlations between QQS and CBI, SIB, JSS and turnover intention confirming that the concurrent validity of the QQS was great. Cronbach's alpha and McDonald's omega of the QQS were 0.803 and 0.806 respectively.

**Conclusion:**

QQS, a three-factor nine-item scale, has robust psychometric properties. QQS is an easy-to-administer, brief, reliable and valid tool to measure employees' quiet quitting. We recommend the use of the QQS in different societies and cultures to assess the validity of the instrument.

## 1. Introduction

A viral TikTok video posted on July 25, 2022 has brought significant mainstream media attention to the phenomenon of “quiet quitting” [1]. Quiet quitting involves employees, and though the term is new, it is not entirely a new phenomenon [2]. Quiet quitting is a work-related phenomenon where employees do not literally quit their jobs but intentionally limit their work, just doing the bare minimum [1]. Furthermore, employees do not volunteer to perform additional tasks and they do not go above and beyond what is expected. Also, quiet quitting can threaten employees' productivity [3]. There are differences between quiet quitting and quitting. In the case of quiet quitting, employees remain at their jobs but are frustrated, have lost their passion for their job and try to work as little as possible. In contrast, in the case of quitting, employees have already left their jobs and are looking for a new workplace. In other words, quitting refers to workers who have already quit their work, while “quiet quitting” is a softer approach since workers do not quit their jobs but they adopt poor work behavior by not exceeding their baseline obligations. Possibly, quiet quitting can be considered as a precursor of employees' turnover. It is quite likely that employees that experience high levels of quiet quitting in the future will leave their jobs since they consider their workplace culture as poor. Thus, quiet quitting can be a predictor of quitting among employees. In this context, measuring quiet quitting with valid tools is crucial to understand deeply this phenomenon.

Furthermore, a valid tool to measure the phenomenon of quiet quitting allows us to identify quiet quitters in a workplace. Afterwards, organizations, policy makers and managers can develop and implement interventions to reduce quiet quitting in order avoiding employees' turnover in the future. In other words, understanding of quiet quitting allows organizations and managers to achieve a sufficient management and improve workplace culture.

The quiet quitting trend follows the great resignation phenomenon that occurred amid the COVID-19 pandemic [4]. Financial difficulties, especially after the pandemic, force workers to stay at their works, but nowadays they decide to prioritize their personal life instead of their career in order to achieve a better work-life balance [5]. Workers adopt now the trend of quiet quitting by responding insufficient to work demands. Several scholars have already recognized that quiet quitting is a significant threat for work productivity by triggering a toxic workplace culture [2],[3],[6]–[8]. In this context, employees' work attitudes could be altered and disrupted resulting in dissatisfaction, disengagement, and turnover intention. Furthermore, high levels of quiet quitting among workers in specific industries such as healthcare industry can lead to a decline in the work productivity threatening patients quality of care and increasing healthcare cost [1],[5],[9],[10]. Thus, identification of quiet quitters among healthcare workers is essential to reduce their disengagement and dissatisfaction and improve their passion to provide high quality healthcare. Therefore, a valid tool to measure quiet quitting among workers especially in specific domains (e.g., healthcare workers) is necessary to develop the appropriate strategies in order to change work attitudes and behaviors among quiet quitters and, thus, improve public health.

Although quiet quitting helps workers avoid burnout, it may compromise their professional careers [6]. Moreover, COVID-19 pandemic causes drastic changes in workplaces that are likely to increase quite quitting among employees [3]. Literature suggests that prevalence of quiet quitting has increased dramatically after the end of COVID-19 lockdowns [11]–[13]. For example, a recent survey conducting in the USA during the COVID-19 pandemic found that half of the participants are considered quiet quitters [11]. Also, another study found that 80% of quiet quitters were burnt out [13]. Since the COVID-19 pandemic caused a tremendous increase on workers' burnout [14]–[16], an increase also in the prevalence of quiet quitting seems to be reasonable. Moreover, literature supports a negative relationship between burnout and work engagement during and after the COVID-19 pandemic [17].

Several instruments have been developed until now to measure work-related variables, such as job burnout, job satisfaction, turnover intention, work engagement, work overload, etc. [18]–[22]. Most of these instruments are reliable and valid and have been translated in several languages, i.e., Maslach burnout inventory, generic job satisfaction scale, job satisfaction survey, Copenhagen burnout inventory, Utrecht work engagement scale, etc. All these instruments measuring work-related variables are multi-dimensional concepts. For example, the Maslach burnout inventory measures three dimensions of the job burnout: exhaustion, cynicism/detachment and professional inefficacy [22]. Additionally, the Bergen burnout inventory assesses exhaustion at work, cynicism and the sense of inadequacy at work [23], while the Utrecht work engagement scale consists of three scales measuring vigor, dedication and absorption [24]. Therefore, we expected that a scale measures the phenomenon of quiet quitting would also be a multi-dimensional concept since quiet quitting is a work-related concept. In this context, we considered a priori several dimension structures for our scale taking into consideration structures in similar instruments that measure work-related variables [18]–[24] and the theoretical concept of quiet quitting [1],[2],[7],[8]. Therefore, we considered the following structures for our scale: (a) detachment, since it is considered as emotional separation from work-related worries and thoughts; (b) lack of motivation, since motivation is considered as individuals' internal disposition and external incentive toward work and (c) lack of initiative, since initiative refers to the ability that workers have to go above and beyond in their work and do more than what is asked by their supervisors. Additionally, we considered that several other structures such as emotional exhaustion, depersonalization, lack of personal accomplishment, exhaustion at work, job satisfaction, work-related burnout and client-related burnout are not compatible with the concept of quiet quitting.

However, it is interesting to note that no instrument is developed to measure the phenomenon of quiet quitting among employees. Thus, developing a valid and reliable instrument to measure employees' quiet quitting is both timely and important. Given that quiet quitting is an alarming issue that has not been investigated in depth and the lack of a specific tool to measure this concept, we aimed to develop a reliable and valid instrument to assess quiet quitting among employees.

## 2. Materials and Methods

### 2.1. Development of the scale

**Figure 1.**
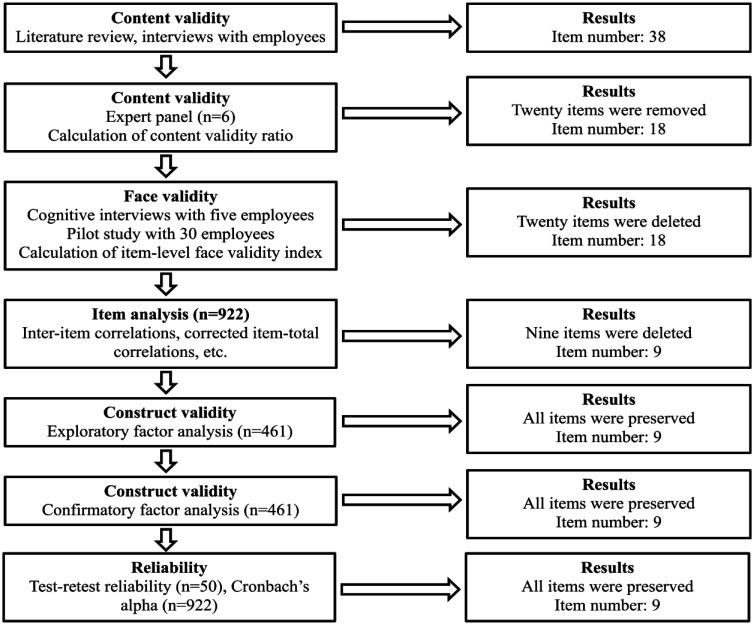
Development and validation of the quiet quitting scale.

We developed the quiet quitting scale (QQS) according to the steps that literature suggests [25]. Development and validation of the QQS are shown in Figure 1. First, we conducted a complete and thorough literature review to assess scales on work-related burnout, stress and satisfaction. In particular, we first identified reviews on scales measuring job burnout, stress and satisfaction [18],[21],[26]–[30]. Afterward, we identified the scales that measure job burnout, stress and satisfaction, e.g. Maslach burnout inventory [22], Copenhagen burnout inventory [31], job satisfaction survey [32], single item burnout scale [33], generic job satisfaction scale [34], Utrecht work engagement scale [24], etc. Finally, we discussed the way that the items of these scales can be useful to develop the items of the QQS. Also, we conducted interviews with six employees from different areas of the labor-market: three from the public sector and three from the private sector. These employees have been working in companies, healthcare services, hospitality services, schools and public services. We encouraged employees to give comments concerning the phenomenon of quiet quitting. Moreover, we discussed with them the items that we have created after the systematic review we performed in the previous step. Afterward, we created a list of 38 items.

Second, an expert panel evaluated the 38 items. Expert panel comprised a psychologist, a psychiatrist, a health psychologist, a general physician, a social worker, and a sociologist. Experts evaluated how well the 38 items correspond to quiet quitting. In particular, experts rated each item as “not essential”, “useful but not essential” or “essential”. Afterward, we calculated the content validity ratio and items with a value >0.80 were remained in our scale [35]. We deleted 20 items based on the ratings from the expert panel. Thus, 18 items remained.

Third, we performed cognitive interviews with five employees and they interpreted the 18 items as we intended [36]. We did not make changes on this step since the five employees indicated no changes were needed.

Finally, we conducted a pilot study with 30 employees to obtain an initial assessment of the scale and assess the face validity. Fifteen employees were females and 15 were males with a mean age of 42.4 years (standard deviation=11.3) and a mean number of work experience of 17.3 years (standard deviation=11.6). We requested employees to rate the clarity of the 18 items based on a four-point Likert scale (1=item is not clear, 2 = item is somewhat clear, 3 = item is quite clear and 4 = item is highly clear). We calculated the item-level face validity index, i.e., the percentage of employees giving an item a clarity rating of three or four. Items with an item-level face validity index >0.80 remained in our scale [37]. Face validity index ranged from 0.833–0.967 for the 18 items. Thus, face validity of the 18 items was excellent and we kept these items through this step.

Eleven items (for example, “I feel detached from my job”, “I feel inspired when I work” and “I feel isolated at work”) were rated on a five-point Likert scale as follows: (1) strongly disagree, (2) disagree, (3) neither disagree or agree, (4) agree and (5) strongly agree. Also, seven items (for example, “How often do you take initiative at your work?” and “How often do you answer phone calls/messages (mail, sms, viber, messenger) from your work after your work shift?”) were rated on a five-point Likert scale as follows: (1) never, (2) rarely, (3) sometimes, (4) often, (5) always. Higher values indicated higher levels of quiet quitting. Seven items were reverse-scored (i.e., “I give my best at work”, “I find motives in my job”, “I feel inspired when I work”, “How often do you take initiative at your work?”, “How often do you help your colleagues when you have completed your own tasks?”, “How often do you answer phone calls/messages (mail, sms, viber, messenger) from your work after your work shift?” and “How often do you answer phone calls/messages (mail, sms, viber, messenger) from your work on a day off?”).

### 2.2. Participants and procedure

Our target population comprises employees in public and private sector in Greece. This study's participants were adults aged 18 years or older working at public and private sector across Greece and being able to understand Greek. We recruited participants from face-to-face interviews, social media (i.e., Facebook, Instagram, Viber and WhatsApp), blogs, e-mail campaigns and SMS campaigns. Thus, a convenience sample was obtained. We collected data during June 2023.

Final overall sample consisted of 922 employees. We measured employees' basic demographic information, i.e., gender, educational level, age, job sector and work experience.

### 2.3. Item analysis

Then, we performed an item analysis for the 18 items that were produced after the initial development phase. In particular, in the overall sample, we checked inter-item correlations, corrected item-total correlations, floor and ceiling effects, skewness, kurtosis and Cronbach's alpha (when a single item was deleted) for the 18 items [38]. Acceptable values for inter-item correlation ranged from 0.15 to 0.75 [39] and ≥0.30 for item-total correlation [40]. If more than 15% of employees achieved the lowest or highest possible score, we considered floor or ceiling effects to be present, respectively [41]. The commonly used 15% threshold was adopted to identify floor or ceiling effects in order to avoid substantial proportions of participants to be near the minimum or maximum value respectively on the five-point Likert scale of the QQS. Examination of floor and ceiling effects is necessary to avoid attenuation or inflation in median and mean estimates and attenuation in variance estimates [42],[43]. Skewness and kurtosis values from -1 to +1 indicated the presence of normal distribution. After these statistical analyses, we examined retained and excluded items in a theoretical basis. In particular, we checked if it was appropriate to remove the items that were deleted after item analysis.

### 2.4. Construct validity

We performed exploratory factor analysis (EFA) and confirmatory factor analysis (CFA) to define the various factors that define the QQS. Since the overall sample comprised 922 employees, we randomly divided them into two groups in order to use different employees for EFA and CFA. Thus, sample for EFA included 461 employees and sample for CFA included 461 employees. Minimum sample size for EFA requires at least 50 observations [44] or five observations per item [38]. Moreover, CFA requires at least 200 observations [45]. Our sample was bigger than these minimum requirements.

First, we conducted EFA to identify the underlying factor structure of the QQS. Then. we conducted CFA to verify the results of EFA. In this step, we analyzed the nine items that were emerged after the initial development of the QQS and the item analysis.

We calculated the Kaiser-Meyer-Olkin index and p-value for Bartlett sphericity test to check the applicability of the EFA. Acceptable values were >0.80 for the Kaiser-Meyer-Olkin index and <0.05 for Bartlett sphericity test. Since we expected that possible factors that were developed during the EFA would be correlated, we used oblique rotation (promax method in SPSS). We applied the following acceptable values: eigenvalues greater than 1, factor loadings greater than 0.60, communalities greater than 0.40 and the total variance explained by the factors >65% [46]. Moreover, we calculated Cronbach's alpha for the factors that were produced by the EFA. The acceptable level of Cronbach's alpha was >0.7 [47]. Finally, we named the factors rationally and appropriately.

Then, we conducted CFA to further test the construct validity of the QQS. We performed CFA to confirm the validity of the QQS factor structure. Since the scale was normally distributed, we used the maximum likelihood estimator. We checked the goodness of fit indices in CFA by calculating two indices of absolute fit, two indices of relative fit and one index of parsimonious fit. In particular, we calculated root mean square error of approximation (RMSEA) and goodness of fit index (GFI) as absolute fit indices, normed fit index (NFI) and comparative fit index (CFI) as relative fit indices and chi-square/degree of freedom (x2/df) as a parsimonious fit index. We used the following acceptable values: RMSEA < 0.10 [48],[49], GFI > 0.90 [50], NFI > 0.90 [49], CFI > 0.90 [49] and x2/df < 5 [41]. Moreover, we calculated standardized regression weights between items and factors and correlation coefficients between factors.

### 2.5. Concurrent validity

We checked the concurrent validity of the QQS on the overall sample (*n* = 922). In that case, we used the job satisfaction survey (JSS) [32], the copenhagen burnout inventory (CBI) [31], the single item burnout (SIB) measure [33] and a single item to measure turnover intention [51]. The JSS comprises 36 items, and total score ranges from 36 to 216 with higher scores indicate higher job satisfaction. JSS is a reliable and valid tool in Greek [52]. In our study, Cronbach's alpha for the JSS was 0.878. The CBI comprises three factors: personal burnout (six items), work-related burnout (seven items) and client-related burnout (six items). Each factor takes values from 0 to 100, and higher scores indicate higher levels of burnout. CBI has been validated in Greek [53]. In our study, Cronbach's alpha for the factor “personal burnout” was 0.919, 0.878 for the factor “work-related burnout” and 0.860 for the factor “client-related burnout”. The SIB measures overall work burnout on a scale from 0 (i.e., not at all burnt out) to 10 (i.e., extremely burnt out). SIB is a reliable and valid tool in Greek [54]. We measured turnover intention with the question “How often have you seriously considered leaving your current job?” where answers are on a six-point Likert scale (i.e., never, rarely, sometimes, somewhat often, quite often and extremely often).

We measured a total score for the QQS and the factors that were created from the factor analysis. In particular, we summed the answers in all items and divided the aggregate by the total number of items in order to calculate the total score for the QQS. Similarly, we calculated the score for each factor. All scores ranged from one to five, and higher scores indicated higher levels of quiet quitting.

We expected a negative relationship between the QQS and the JSS but a positive relationship between the QQS and the CBI, the SIB measure and the turnover intention.

### 2.6. Reliability

First, we checked the reliability of the QQS on the overall sample (*n* = 922). In that case, we measured Cronbach's alpha and McDonald's omega. The acceptable level of Cronbach's alpha and McDonald's Omega was >0.7 [47]. Moreover, we calculated corrected item-total correlations, inter-item correlations and Cronbach's alpha when a single item was deleted for the nine items of the final structure model of the QQS. Acceptable values for inter-item correlation ranged from 0.15–0.75 [39] and for item-total correlation were ≥0.30 [40].

Furthermore, we conducted a test-retest study with 50 employees who completed the QQS twice in two weeks. We calculated Cohen's kappa for the nine items since the answers were in an ordinal scale. Also, we measured intraclass correlation coefficient for the total score of QQS and for scores on three factors. In particular, we calculated the two-way mixed intraclass correlation coefficient (absolute agreement).

### 2.7. Ethical considerations

Our study protocol was approved by the Ethics Committee of the Faculty of Nursing, National and Kapodistrian University of Athens (approval number; 451, June 2023). We did not collect personal data, and we obtained informed consent of the employees. Moreover, participation was anonymous and voluntary. We conducted our study in accordance with the Declaration of Helsinki [55].

### 2.8. Statistical analysis

We used AMOS version 21 (Amos Development Corporation, 2018) to conduct CFA using. All other analyses were conducted with IBM SPSS 21.0 (IBM Corp. Released 2012. IBM SPSS Statistics for Windows, Version 21.0. Armonk, NY: IBM Corp.). We used absolute numbers and percentages to present categorical variables. Also, we used mean, standard deviation, median, minimum value and maximum value to present continuous variables. We used chi-square test, chi-square trend test and independent samples t-test to compare the two groups of employees regarding EFA and CFA. We used Pearson's correlation coefficient to assess correlation between QQS and JSS, CBI and SBI since all scales followed normal distribution. Also, we used Spearman's correlation coefficient to assess correlation between QQS and turnover intention since intention was measured on an ordinal scale. P-values less than 0.05 were considered as statistically significant.

## 3. Results

### 3.1. Employees' characteristics

Our sample included 922 employees. Among them, we used 461 employees to perform EFA and 461 employees to perform CFA. We present demographic characteristics of our sample in Table 1. The mean age of the employees was 40.8 years (*SD* = 9.2), with an age range of 22–74 years. The majority of the employees were females (72%). Mean work experience was 16.5 years (*SD* = 9.3), with a range of 4–40 years. Regarding employment patterns, 50.8% of the employees had been working in the public sector, and 49.2% in the private sector. Further, most of the participants had a university degree (89.6%), and among them, 54.0% also possessed a MSc/PhD diploma. We did not find differences between EFA and CFA employees (*p* = 0.08 for gender, *p* = 0.56 for educational level, *p* = 0.56 for age, *p* = 0.09 for job sector and *p* = 0.89 for years of experience).

**Table 1.** Demographic characteristics of employees (*N* = 922).

**Characteristics**	**Total (*n* = 922)**		**EFA (*n* = 421)**		**CFA (*n* = 421)**		***P*-value**
	***n***	**%**	***n***	**%**	***n***	**%**	
Gender							0.08^a^
Females	664	72.0	344	74.6	320	69.4	
Males	258	28.0	117	25.4	141	30.6	
Educational level							0.56^b^
High school	96	10.4	52	11.3	44	9.5	
University degree	328	35.6	150	32.5	178	38.6	
MSc/PhD diploma	498	54.0	259	56.2	239	51.8	
Age^c^	40.8	9.2	41.0	9.2	40.6	9.3	0.56^d^
Job sector							0.09^a^
Private	454	49.2	214	46.4	240	52.1	
Public	468	50.8	247	53.6	221	47.9	
Years of experience^c^	16.5	9.3	16.5	9.3	16.4	9.4	0.89^d^

Note: ^a^ chi-square test; ^b^ chi-square trend test; ^c^ mean, standard deviation; ^d^ independent samples t-test.

### 3.2. Item analysis

Descriptive statistics, corrected item-total correlations, floor and ceiling effects, skewness, kurtosis and Cronbach's alpha (when a single item was deleted) for the 18 items that were produced after the initial development phase are shown in Table 2, while inter-item correlations are shown in Supplementary Table 1. First, we deleted items #14 and #15 due to negative inter-item correlations. Then, we deleted item #16 due to low corrected item-total correlation and low inter-item correlations with ten other items. Next, we deleted items #1, #4 and #17 due to high floor and ceiling effects, skewness and kurtosis. Also, we deleted items #2, #6 and #13 due to low inter-item correlations with six other items. Then, we examined the meaning of excluded and retained items on a theoretical basis in order to judge the results of the item analysis. For all the items that were removed there were items that remained and had similar meaning. For example, item #1 had a similar meaning with the items #3 and #5, the item #2 with the items #7 and #10 and the item #4 with the items #8 and #9. Thus, the removal of the nine questions is also justified in a theoretical context.

Thus, we deleted nine items. The remaining nine items (i.e., #3, #5, #7, #8, #9, #10, #11, #12 and #18) had acceptable corrected item-total correlations, inter-item correlations, floor and ceiling effects, skewness and kurtosis. Moreover, Cronbach's alpha for the 18 items was 0.85 and was decreased after elimination of each single item.

**Table 2.** Descriptive statistics, corrected item-total correlations, floor and ceiling effects, skewness, kurtosis and Cronbach's alpha (when a single item was deleted) for the 18 items that were produced after the initial development phase (*N* = 922).

**Item**	**Mean (standard deviation)**	**Corrected item-total correlation**	**Floor effect (%)**	**Ceiling effect (%)**	**Skewness**	**Kurtosis**	**Cronbach's alpha if item deleted**	**Item exclusion or retention**
1. I give my best at work.	1.46 (0.77)	0.376	2.0	64.9	2.43	7.42	0.841	Excluded
2. I feel detached from my job.	2.51 (1.11)	0.525	20.4	4.3	0.36	-0.65	0.834	Excluded
3. I find motives in my job.	2.68 (1.12)	0.461	6.5	14.8	0.32	-0.66	0.837	Retained
4. I don't care about my job.	1.71 (0.91)	0.606	52.1	1.5	1.38	1.73	0.831	Excluded
5. I feel inspired when I work.	2.67 (1.04)	0.527	5.6	12.4	0.33	-0.30	0.834	Retained
6. I feel isolated at work.	2.15 (1.09)	0.400	34.5	3.0	0.73	-0.29	0.840	Excluded
7. I do the basic or minimum amount of work without going above and beyond.	1.86 (1.03)	0.632	46.4	3.5	1.30	1.27	0.829	Retained
8. If a colleague can do some of my work, then I let him/her do it.	2.38 (1.11)	0.387	24.7	3.5	0.46	-0.63	0.841	Retained
9. I don't express opinions and ideas about my work because I am afraid that the manager assigns me more tasks.	2.05 (1.07)	0.551	37.7	2.4	0.86	-0.07	0.833	Retained
10. I don't express opinions and ideas about my work because I think that working conditions are not going to change.	2.37 (1.22)	0.486	29.3	6.1	0.56	-0.70	0.836	Retained
11. I take as many breaks as I can.	2.16 (1.05)	0.556	31.0	2.8	0.74	-0.08	0.833	Retained
12. How often do you take initiative at your work?	2.23 (0.90)	0.488	1.7	19.7	0.63	0.37	0.836	Retained
13. How often do you help your colleagues when you have completed your own tasks?	1.93 (0.86)	0.303	1.5	32.8	1.02	1.40	0.844	Excluded
14. How often do you answer phone calls/messages (mail, sms, viber, messenger) from your work after your work shift?	2.22 (1.17)	0.315	5.2	33.8	0.72	-0.34	0.845	Excluded
15. How often do you answer phone calls/messages (mail, sms, viber, messenger) from your work on a day off?	2.41 (1.28)	0.333	9.8	30.2	0.62	-0.65	0.845	Excluded
16. How often do you go to work later and/or leave work early?	2.09 (1.11)	0.226	38.4	3.9	0.83	-0.03	0.849	Excluded
17. How often do you take sick leave even though you can work?	1.43 (0.80)	0.388	72.0	1.3	2.17	4.95	0.841	Excluded
18. How often do you pretend to be working in order to avoid another task?	1.69 (0.92)	0.526	54.9	0.9	1.30	1.08	0.835	Retained

### 3.3. Exploratory factor analysis

We conducted EFA including the nine items (i.e., #3, #5, #7, #8, #9, #10, #11, #12 and #18) mentioned above. In that case, we used the first subsample of 461 employees. We found acceptable values for Kaiser-Meyer-Olkin index (0.845) and p-value for Bartlett sphericity test (<0.001). We performed oblique rotation and we found three factors including all items (Table 3). Factor loadings ranged from 0.651–0.852, while communalities ranged from 0.519–0.756. We named the three factors as follows: detachment (items #7, #8, #11, #18), lack of initiative (items #9, #10, #12) and lack of motivation (items #3, #5). The total variance explained by the three factors was 65.028%. Cronbach's alpha for the total scale was 0.803, 0.707 for the factor “detachment”, 0.706 for the factor “lack of initiative” and 0.747 for the factor “lack of motivation”. Thus, EFA identified a three-factor model with nine items.

As we mentioned in the introduction, we hypothesized a three-factor model for the QQS. EFA confirmed our hypothesis since we identified three factors, i.e., “detachment”, “lack of initiative” and “lack of motivation”. The factor “detachment” included the items #7, #8, #11 and #18 (Table 3), which referred to individuals' tendency to separate themselves from work-related worries and thoughts. Additionally, the factor “lack of initiative” included the items #9, #10 and #12, which measured individuals' tendency to take initiative and go above and beyond in their work. Moreover, the factor “lack of motivation” included two items, which referred to individuals' internal disposition (#5) and external incentive (#3) toward work.

### 3.4. Confirmatory factor analysis

**Figure 2.**
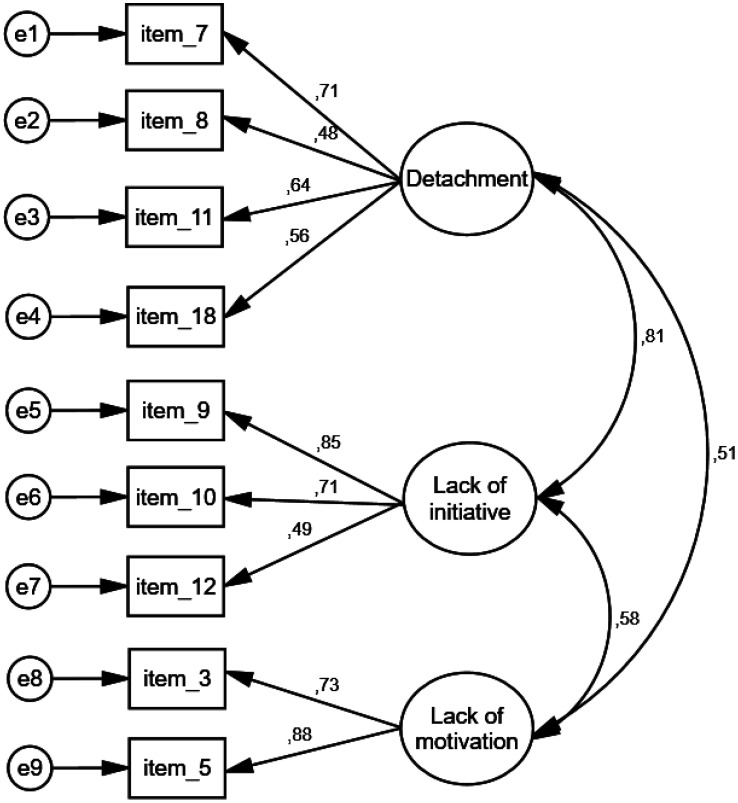
Confirmatory factor analysis of the quiet quitting scale.

Then, we used the second subsample (*n* = 461) to conduct the CFA. We performed CFA to verify the factors obtained from EFA. Thus, we performed CFA of nine items across three factors. The goodness-of-fit statistics suggested that the 3-factor model with nine items of the QQS provided a very good fit to data; x^2^/df = 3.184, RMSEA = 0.069 (90% confidence interval = 0.052 to 0.087), GFI = 0.964, NFI = 0.939, CFI = 0.957. Moreover, standardized regression weights between items and factors ranged from 0.478 to 0.878 (p < 0.001 in all cases). Furthermore, the correlations between the three factors were positive and statistically significant (p < 0.001 in all cases). CFA of the QQS is shown in Figure 2. Correlation between the factors “detachment” and “lack of initiative” was strong (*r* = 0.81), while between the factors “detachment” and “lack of motivation” and the factors “lack of initiative” and “lack of motivation” was moderate (*r* = 0.51 and *r* = 0.58) respectively.

In conclusion, EFA and CFA identified a three-factor nine-item model for the QQS: detachment (four items), lack of initiative (three items) and lack of motivation (two items), (Supplementary Table 2).

**Table 3.** Exploratory factor analysis using oblique rotation (promax method) for the “Quiet Quitting” scale (*n* = 461).

**Items**	**Factors**			**Communalities**
	**Detachment (items #7, #8, #11, #18)**	**Lack of initiative (items #9, #10, #12)**	**Lack of motivation (items #3, #5)**	
3. I find motives in my job.	0.080	0.153	0.852	0.756
5. I feel inspired when I work.	0.196	0.129	0.811	0.713
7. I do the basic or minimum amount of work without going above and beyond.	0.651	0.401	0.267	0.656
8. If a colleague can do some of my work, then I let him/her do it.	0.695	0.213	-0.221	0.578
9. I don't express opinions and ideas about my work because I am afraid that the manager assigns me more tasks.	0.288	0.780	-0.008	0.692
10. I don't express opinions and ideas about my work because I think that working conditions are not going to change.	-0.007	0.834	0.184	0.729
11. I take as many breaks as I can.	0.741	0.123	0.214	0.610
12. How often do you take initiative at your work?	0.192	0.679	0.147	0.519
18. How often do you pretend to be working in order to avoid another task?	0.731	0.021	0.256	0.600

Note: Values express factors loadings.

### 3.5. Concurrent validity

We found a negative relationship between the QQS and the JSS, suggesting that employees with lower levels of job satisfaction may have higher levels of quiet quitting. Correlation coefficients between QQS and JSS ranged from -0.28 to -0.54 (p < 0.001 in all cases). Moreover, we found a positive relationship between the QQS and the CBI, the SIB measure and the turnover intention (p < 0.001 in all cases). Correlation coefficients between QQS and CBI subscales ranged from 0.21–0.47. A similar pattern was observed in case of SBI (correlation coefficients ranged from 0.17–0.34). Also, correlation coefficients between the QQS and turnover intention were even higher (0.30–0.48). Therefore, the concurrent validity of the QQS was very good. Correlation analysis between the scales is shown in Table 4.

**Table 4.** Correlations between the quiet quitting scale (QQS) and the job satisfaction survey (JSS), the Copenhagen burnout inventory (CBI), the single item burnout (SIB) measure and the turnover intention (*n* = 922).

**QQS**	**JSS**		**CBI**						**SIB**		**Turnover intention**	
			**Work-related burnout**		**Personal burnout**		**Client-related burnout**					

	**PCC**	**P-value**	**PCC**	**P-value**	**PCC**	**P-value**	**PCC**	**P-value**	**PCC**	**P-value**	**SCC**	**P-value**
Detachment	-0.28	<0.001	0.30	<0.001	0.26	<0.001	0.21	<0.001	0.20	<0.001	0.46	<0.001
Lack of initiative	-0.33	<0.001	0.35	<0.001	0.31	<0.001	0.26	<0.001	0.17	<0.001	0.30	<0.001
Lack of motivation	-0.54	<0.001	0.47	<0.001	0.37	<0.001	0.36	<0.001	0.34	<0.001	0.35	<0.001
Total score	-0.46	<0.001	0.45	<0.001	0.39	<0.001	0.34	<0.001	0.29	<0.001	0.48	<0.001

Note: PCC: Pearson's correlation coefficient; SCC: Spearman's correlation coefficient.

### 3.6. Reliability

Reliability analysis included the nine items that emerged from the factor analysis. McDonald's omega and Cronbach's alpha are shown in Table 5. All values were higher than 0.70, indicating acceptable internal consistency of the QQS. Cronbach's alpha of the QQS was 0.803 and McDonald's omega was 0.806. Cronbach's alpha of the factors “detachment”, “lack of initiative” and “lack of motivation” was 0.707, 0.706 and 0.747 respectively. Also, McDonald's omega of the factors “detachment” and “lack of initiative” was 0.711 and 0.735 respectively. Moreover, corrected item-total correlations had values between 0.363 and 0.616, while all inter-item correlations had values between 0.15 and 0.70. Also, removal of each single item did not increase Cronbach's alpha (Supplementary Table 3). Range of Cronbach's alpha when a single item was deleted was 0.768–0.801.

Cohen's kappa for the nine items ranged from 0.836–0.945 (p < 0.001 in all cases), (Supplementary Table 4). Moreover, intraclass correlation coefficient for the total score was 0.993, while for the three factors ranged from 0.987–0.992 (p < 0.001 in all cases), (Supplementary Table 5). Therefore, reliability of QQS was excellent.

Moreover, less than 15% of employees achieved the lowest or highest possible score of the total and factors scores of the QQS indicating that the instrument is reliable.

**Table 5.** McDonald's omega and Cronbach's alpha for the three-factor model with nine items for the quiet quitting scale.

	**Cronbach's alpha**	**McDonald's Omega**
Detachment	0.707	0.711
Lack of initiative	0.706	0.735
Lack of motivation	0.747	NC
Total score	0.803	0.806

Note: NC: noncomputable due to limited number of items.

## 4. Discussion

Due to recent findings by survey firm Gallup, about half of USA workers are quiet quitting their job [11]. The phenomenon of quiet quitting has been around even before the COVID-19 pandemic, but after pandemic, its prevalence has increased dramatically [12]. COVID-19 pandemic causes a tremendous impact on working conditions, including the wide adoption of remote working, job insecurity, unemployment and flexible work arrangements [3]. Additionally, the post-COVID-19 period was marked by a large number of resignations [4],[6]. Therefore, organizations, managers and employers should give particular attention to quiet quitting, since it may change employees behaviors towards their jobs and affect the relationship between employees and their employers [56].

Thus, it is important to assess the phenomenon of quiet quitting with valid and reliable instruments. Although there are several instruments in the literature examining work-related variables, such as turnover intention, job burnout and job satisfaction [18]–[21], we did not find an instrument to measure quiet quitting. Given the research gap, we developed and validated a new specific measurement tool to determine quite quitting among employees.

In this study, we presented the development and validation of a new scale, namely quiet quitting scale. We found that the QQS has a stable three-factor structure. Moreover, the QQS is a brief and easy to administer instrument since it comprises only nine items. Initial psychometric analysis demonstrated that the QQS had very good validity and excellent reliability. Our development procedure and validation analysis seem to be robust since we examined several types of validity and reliability as literature suggests, e.g., content validity, face validity, item analysis, construct validity, test-retest reliability, etc. [25],[37]–[39],[41]. Thus, we applied a rigorous methodological approach, which complies with the reliability and validity stages of the QQS.

In our study, the higher the score on the QQS, the higher the scores on the CBI, the SIB measure and the turnover intention were. Also, we found a negative relationship between the QQS and the JSS. Thus, the high concurrent validity allows us to conclude that higher scores on the QQS indicate higher levels of quiet quitting. Therefore, the overall score of the summed-up items answers can indicate the level of quiet quitting among employees. Moreover, we found the same pattern for the three factors of the QQS: detachment, lack of initiative and lack of motivation. Additionally, the significant correlations between the QQS and work-related burnout (on the CBI and the SIB measure) and job satisfaction (on the JSS) suggest that employees with high levels of quite quitting may have also high levels of work-related burnout and low levels of job satisfaction. This finding is supported by a recent study in the USA where four out of five quiet quitters were burnt out [13]. Moreover, several systematic reviews provide evidence that work-related burnout is associated with job dissatisfaction, work disengagement and turnover intention [57]–[59].

We should note some limitations in our study. First, we employed a convenience sample of employees from the public and private sector in Greece who volunteered to participate in our study. Also, we cannot calculate the response rate since we recruited our sample from sources such as social media, blogs, e-mail campaigns and SMS campaigns. Thus, convenience sampling and unknown response rate weaken the generalizability of our results. However, our sample size meets all criteria for validity and reliability analysis. Therefore, the psychometric analysis is quite powerful. Further studies with random and stratified samples could add significant information. Also, studies with employees from different organizations and companies (e.g., healthcare workers, administrative staff, teachers, academic staff, etc.) could further validate the QQS in more specific study populations. Second, we obtained our data through self-report questionnaires. Thus, information bias could arise because employees' responses could be affected by social desirability factors. Finally, since we assessed the validity of the QQS for first time, we did not attempt to establish a cut-off score that could categorize employees as quiet quitters and non-quite quitters. Future studies could perform cut-off analyses establishing cut-off values in order to discriminate employees.

## 5. Conclusions

According to our findings, the QQS is a 3-factor 9-item scale with robust psychometric properties. Thus, the QQS is a brief, reliable and valid instrument to measure quiet quitting among employees. However, further studies should be conducted to support the reliability and the validity of QQS. We recommend the use of the QQS in different societies and cultures to assess the validity of the instrument. Measuring quiet quitting appropriately could help policy makers to understand this phenomenon and develop and implement measures to diminish its impact on employees' life.

## Use of AI tools declaration

The authors declare they have not used Artificial Intelligence (AI) tools in the creation of this article.
