# Supplementary material for: The quiet quitting scale: Development and initial validation
Source: AIMS Public Health. 2023 Oct 17;10(4):828–48. doi: 10.3934/publichealth.2023055 (PMC10764970; doi:10.3934/publichealth.2023055)
Supplement: Supplementary file 1 [file publichealth-10-04-055-s001.pdf]

Public Health

AIMS Public Health, 10(4): 828–848.
DOI: 10.3934/publichealth.2023055
Received: 20 August 2023
Revised: 10 September 2023
Accepted: 07 October 2023
Published: 17 October 2023

http://www.aimspress.com/journal/aimsph

Research article

The quiet quitting scale: Development and initial validation

Petros Galanis[1,*], Aglaia Katsiroumpa[1], Irene Vraka[2], Olga Siskou[3], Olympia Konstantakopoulou[4], Ioannis Moisoglou[5], Parisis Gallos[6] and Daphne Kaitelidou[4]

[1] Clinical Epidemiology Laboratory, Faculty of Nursing, National and Kapodistrian University of Athens, Athens, Greece
[2] Department of Radiology, P. & A. Kyriakou Children's Hospital, Athens, Greece
[3] Department of Tourism Studies, University of Piraeus, Piraeus, Greece
[4] Center for Health Services Management and Evaluation, Faculty of Nursing, National and Kapodistrian University of Athens, Athens, Greece
[5] Faculty of Nursing, University of Thessaly, Larissa, Greece
[6] Faculty of Nursing, National and Kapodistrian University of Athens, Athens, Greece

* **Correspondence:** Email: pegalan@nurs.uoa.gr; Tel: +302107461471.

Supplementary

Table S1. Inter-item correlations between the 18 items that were produced after the initial development phase (*N* = 922).

Item	2	3	4	5	6	7	8	9	10	11	12	13	14	15	16	17	18
1. I give my best at work.	0.150	0.286	0.286	0.335	0.112	0.288	0.164	0.187	0.111	0.205	0.255	0.165	0.146	0.177	0.143	0.218	0.250
2. I feel detached from my job.		0.396	0.445	0.314	0.494	0.413	0.280	0.308	0.340	0.363	0.263	0.140	0.096	0.046	0.125	0.233	0.316
3. I find motives in my job.			0.382	0.598	0.176	0.290	0.065	0.235	0.301	0.239	0.308	0.121	0.177	0.180	0.083	0.124	0.222
4. I don’t care about my job.				0.401	0.318	0.518	0.320	0.390	0.394	0.389	0.330	0.147	0.127	0.146	0.197	0.249	0.356
5. I feel inspired when I work.					0.163	0.320	0.179	0.300	0.298	0.290	0.305	0.203	0.206	0.197	0.192	0.167	0.267
6. I feel isolated at work.						0.343	0.168	0.325	0.309	0.342	0.183	0.198	0.030	0.027	0.132	0.126	0.194
7. I do the basic or minimum amount of work without going above and beyond.							0.330	0.472	0.379	0.465	0.376	0.148	0.140	0.162	0.224	0.366	0.434
8. If a colleague can do some of my work, then I let him/her do it.								0.331	0.185	0.342	0.166	0.194	0.085	0.145	0.119	0.200	0.302
9. I don’t express opinions and ideas about my work because I am afraid that the manager assigns me more tasks.									0.585	0.352	0.388	0.179	0.099	0.159	0.088	0.178	0.327

Continued on next page

Item	2	3	4	5	6	7	8	9	10	11	12	13	14	15	16	17	18
10. I don’t express opinions and ideas about my work because I think that working conditions are not going to change.										0.271	0.358	0.152	0.129	0.127	0.109	0.094	0.204
11. I take as many breaks as I can.											0.254	0.245	0.163	0.154	0.238	0.295	0.409
12. How often do you take initiative at your work?												0.297	0.282	0.304	0.027	0.100	0.162
13. How often do you help your colleagues when you have completed your own tasks?													0.154	0.164	0.018	0.122	0.151
14. How often do you answer phone calls/messages (mail, sms, viber, messenger) from your work after your work shift?														0.790	-0.074	0.086	0.140
15. How often do you answer phone calls/messages (mail, sms, viber, messenger) from your work on a day off?															-0.036	0.108	0.173
16. How often do you go to work later and/or leave work early?																0.328	0.305
17. How often do you take sick leave even though you can work?																	0.613
18. How often do you pretend to be working in order to avoid another task?																	

Table S2. Final structure of the three-factor nine-item model for the "Quiet Quitting" scale.

**Items**	**Answers**				
	**1**	**2**	**3**	**4**	**5**
1. I do the basic or minimum amount of work without going above and beyond.	Strongly disagree	Disagree	Neither disagree or agree	Agree	Strongly agree
2. If a colleague can do some of my work, then I let him/her do it.	Strongly disagree	Disagree	Neither disagree or agree	Agree	Strongly agree
3. I take as many breaks as I can.	Strongly disagree	Disagree	Neither disagree or agree	Agree	Strongly agree
4. How often do you pretend to be working in order to avoid another task?	Never	Rarely	Sometimes	Often	Always
5. I don't express opinions and ideas about my work because I am afraid that the manager assigns me more tasks.	Strongly disagree	Disagree	Neither disagree or agree	Agree	Strongly agree
6. I don't express opinions and ideas about my work because I think that working conditions are not going to change.	Strongly disagree	Disagree	Neither disagree or agree	Agree	Strongly agree
7. How often do you take initiative at your work?	Never	Rarely	Sometimes	Often	Always
8. I find motives in my job.	Strongly disagree	Disagree	Neither disagree or agree	Agree	Strongly agree
9. I feel inspired when I work.	Strongly disagree	Disagree	Neither disagree or agree	Agree	Strongly agree

Note 1: Factors: detachment (items #1, #2, #3, #4); lack of initiative (items #5, #6, #7), lack of motivation (items #8, #9).

Note 2: Items #7, #8, #9 are reversed-scored.

Note 3: Sum the answers in all items and divide the aggregate by total number of answers in order to calculate the total score for the "Quiet Quitting" Scale. Similarly, calculate the score for each factor. All scores range from one to five. Higher scores indicate higher levels of quiet quitting.

Table S3. Corrected item-total correlations, and Cronbach's alpha when a single item was deleted for the nine items of the final structure model of the "Quiet Quitting" scale.

Items	Corrected item-total correlation	Cronbach's alpha if item deleted
I do the basic or minimum amount of work without going above and beyond.	0.439	0.791
If a colleague can do some of my work, then I let him/her do it.	0.509	0.782
I take as many breaks as I can.	0.616	0.768
How often do you pretend to be working in order to avoid another task?	0.363	0.801
I don't express opinions and ideas about my work because I am afraid that the manager assigns me more tasks.	0.607	0.768
I don't express opinions and ideas about my work because I think that working conditions are not going to change.	0.512	0.782
How often do you take initiative at your work?	0.517	0.781
I find motives in my job.	0.459	0.788
I feel inspired when I work.	0.457	0.788

Table S4. Cohen's kappa for the three-factor model with nine items for the "Quiet Quitting" scale.

Items	Cohen's kappa	*P*-value
1. I do the basic or minimum amount of work without going above and beyond.	0.918	<0.001
2. If a colleague can do some of my work, then I let him/her do it.	0.944	<0.001
3. I take as many breaks as I can.	0.836	<0.001
4. How often do you pretend to be working in order to avoid another task?	0.891	<0.001
5. I don't express opinions and ideas about my work because I am afraid that the manager assigns me more tasks.	0.945	<0.001
6. I don't express opinions and ideas about my work because I think that working conditions are not going to change.	0.897	<0.001
7. How often do you take initiative at your work?	0.915	<0.001
8. I find motives in my job.	0.912	<0.001
9. I feel inspired when I work.	0.912	<0.001

Table S5. Intraclass correlation coefficients for the total score for each factor and the "Quiet Quitting" scale.

"Quiet Quitting" scale	Intraclass correlation coefficient	95% confidence interval	*P*-value
Total score	0.993	0.987 to 0.996	<0.001
Score of the factor "detachment"	0.987	0.977 to 0.993	<0.001
Score of the factor "lack of initiative"	0.992	0.986 to 0.996	<0.001
Score of the factor "lack of motivation"	0.988	0.980 to 0.993	<0.001

AIMS Press

© 2023 the Author(s), licensee AIMS Press. This is an open access article distributed under the terms of the Creative Commons Attribution License (http://creativecommons.org/licenses/by/4.0)
